# Digital data for quick response (QR) codes of alkalophilic *Bacillus pumilus* to identify and to compare bacilli isolated from Lonar Crator Lake, India

**DOI:** 10.1016/j.dib.2016.03.103

**Published:** 2016-04-09

**Authors:** Bhagwan N. Rekadwad, Chandrahasya N. Khobragade

**Affiliations:** School of Life Sciences, Swami Ramanand Teerth Marathwada University, Nanded, Maharashtra 431606, India

**Keywords:** Alkalophiles, Alkaline environment, Bacillus signatures, Lonar Crator Lake, Soda Lake

## Abstract

Microbiologists are routinely engaged isolation, identification and comparison of isolated bacteria for their novelty. 16S rRNA sequences of *Bacillus pumilus* were retrieved from NCBI repository and generated QR codes for sequences (FASTA format and full Gene Bank information). 16SrRNA were used to generate quick response (QR) codes of *Bacillus pumilus* isolated from Lonar Crator Lake (19° 58′ N; 76° 31′ E), India. *Bacillus pumilus* 16S rRNA gene sequences were used to generate CGR, FCGR and PCA. These can be used for visual comparison and evaluation respectively. The hyperlinked QR codes, CGR, FCGR and PCA of all the isolates are made available to the users on a portal https://sites.google.com/site/bhagwanrekadwad/. This generated digital data helps to evaluate and compare any *Bacillus pumilus* strain, minimizes laboratory efforts and avoid misinterpretation of the species.

## **Specifications Table**

**Table:** 

Subject area	*Microbiology*
More specific subject area	*Microbial diversity Informatics*
Type of data	*Text file, sequences, table, figures QR Codes, CGR, FCGR, NJ plot and PCA.*
How data was acquired	*Through NCBI repository*
Data format	*Raw and analyzed*
Experimental factors	*16S rRNA sequences were used for creation of digital information*
Experimental features	*16SrRNA gene sequences were used to create QR codes using DNA BarID software.*
Data source location	Lonar soda lake (19° 58’ N; 76° 31’ E), Buldhana District, India.
Data accessibility	Data is within this article.

**Value of the data**

• Digital information on *Bacillus pumilus* isolated from Lonar Crator Lake has enormous biotechnological applications. Generated digital information appears to be white snow for quick identification and comparison of *Bacillus pumilus*.
• This digitization of 16S RNA sequences of *Bacillus pumilus* from Lonar Crator Lake were carried out first time by us and made available to users.
• This generated digital reduces time and cost on identification and comparison of *Bacillus pumilus*.

## 1 Data

**Fig. 1:**
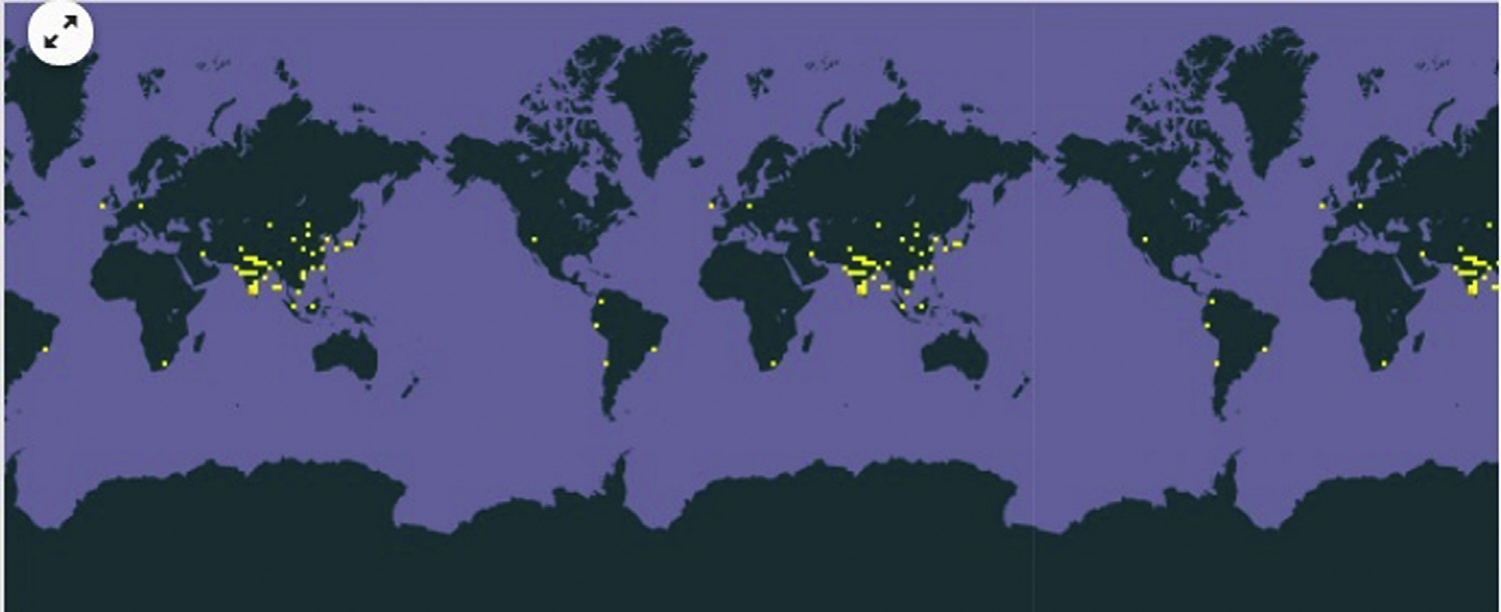
GBIF map for distribution of *Bacillus pumilis*: yellow dots indicate places where researcher who have identified *Bacillus pumilus* strains. Source: GBIF (1).

**Fig. 2:**
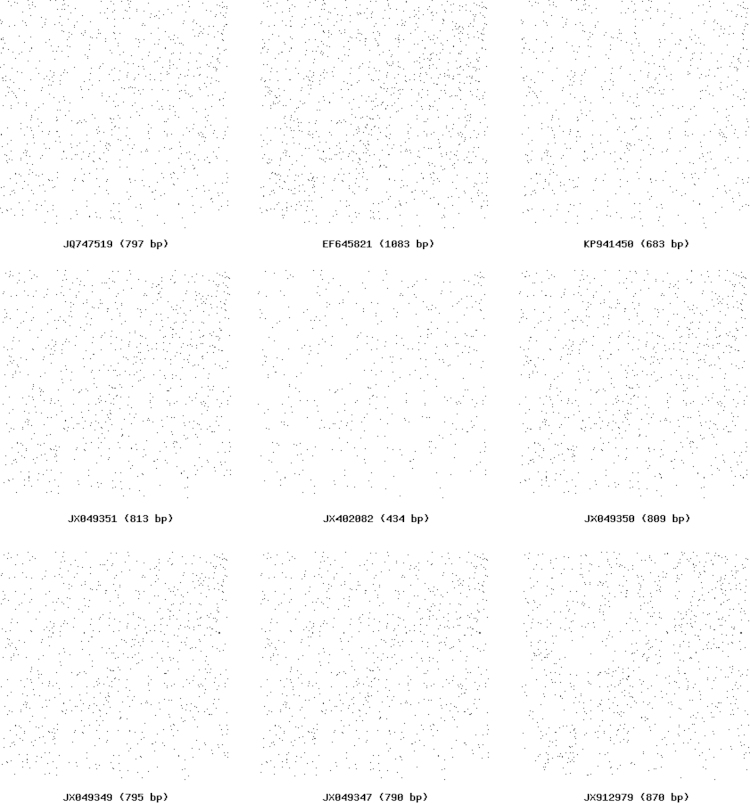

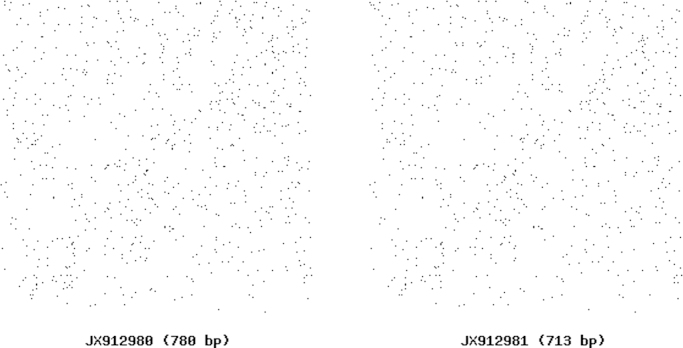
*Bacillus pumilus*: Chaos Game representation (CGR) showing difference in base composition of DNA sequence.

**Fig. 3:**
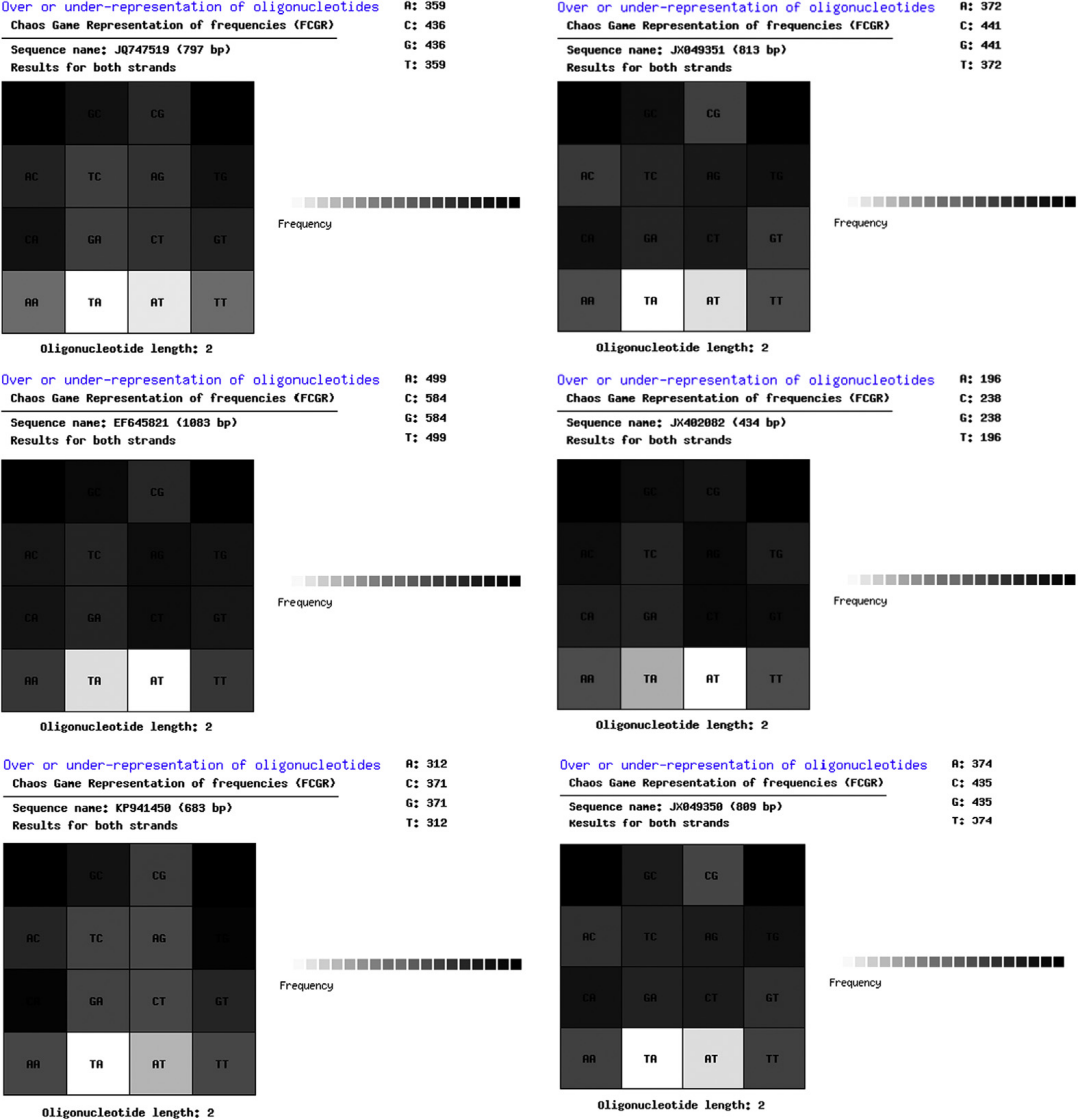

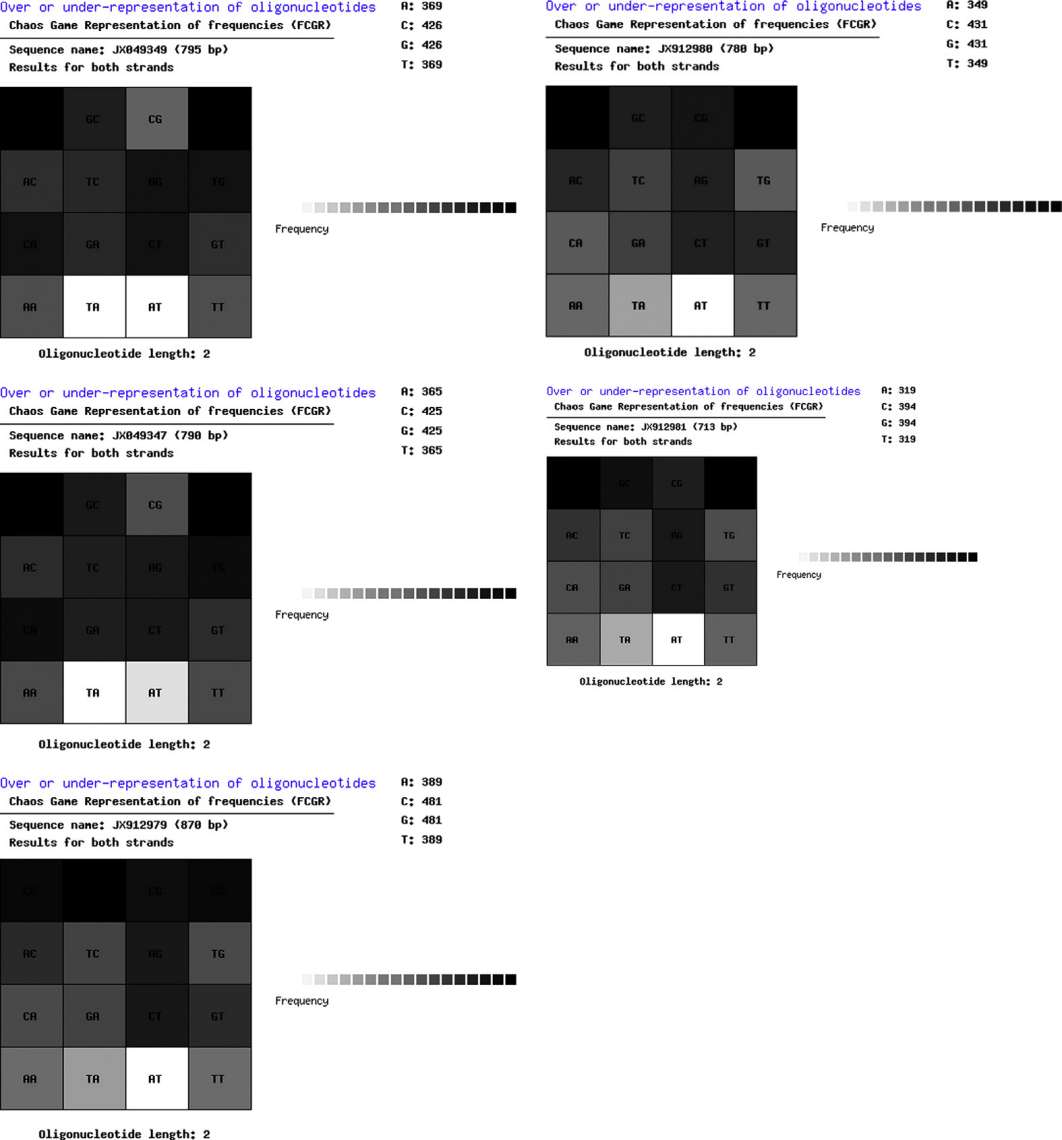
*Bacillus pumilus*: Chaos Game Representation of frequencies (FCGR).

**Fig. 4:**
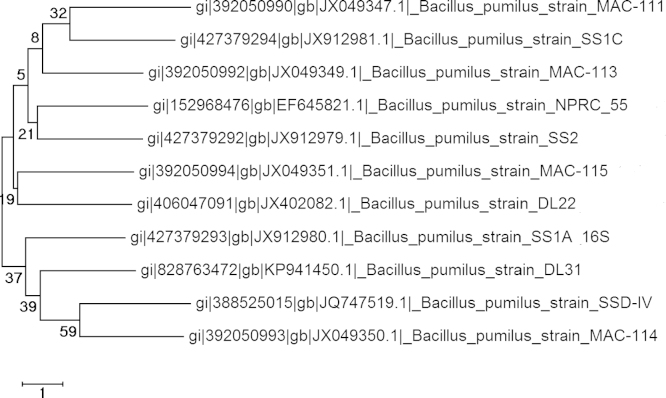
Evolutionary relationships among strains of *Bacillus pumilus* isolated from Lonar Crator Lake, India. The evolutionary history was inferred using the Neighbor-Joining method [9]. The bootstrap consensus tree inferred from 1000 replicates is taken to represent the evolutionary history of the taxa analyzed [10]. Branches corresponding to partitions reproduced in less than 50% bootstrap replicates are collapsed. The evolutionary distances were computed using the Maximum Composite Likelihood method [11] and are in the units of the number of base substitutions per site. The analysis involved 65 nucleotide sequences. All positions containing gaps and missing data were eliminated. There were a total of 591 positions in the final dataset. Evolutionary analyses were conducted in MEGA6 [12].

**Fig. 5:**
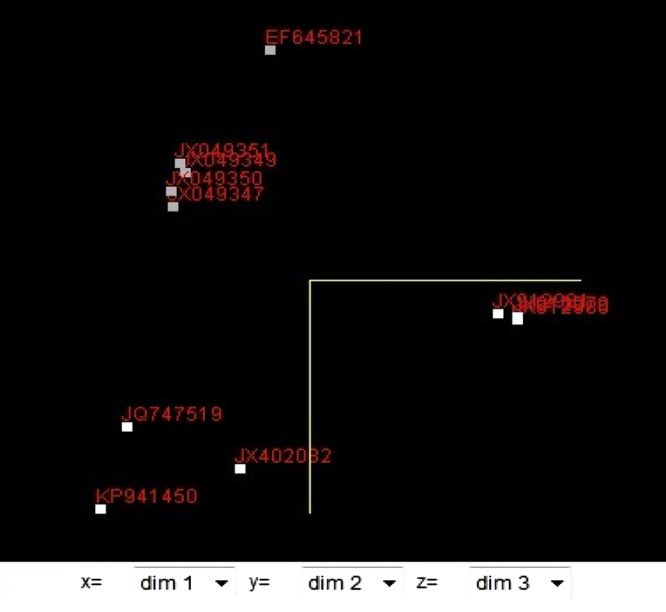
Principal component analysis (PCA) of isolates.

Raw data was obtained through NCBI׳s BioSample database. Digital data of each isolate i.e two quick response (QR) codes, Chaose Game Representation (CGR), Choase Game Representation Frequencies (FCGR) and Principal Component Analysis (PCA) of isolates made available on internet on a portal created by us https://sites.google.com/site/bhagwanrekadwad/ in downloadable format (Table 1, Fig. 1, Fig. 2, Fig. 3, Fig. 4, Fig. 5).

## 2 Experimental design, materials and methods

16S rRNA sequences (full gene bank and FASTA) were retrieved from NCBI BioSample database [1], [2]. DNA BarID downloaded from NEERI-CSIR, Nagpur website [3]. The QR codes for *Bacillus pumilus* from full NCBI Genebank sequences and FASTA sequences were generated which do not resembles with any other species or strains in any database. The generated data were compared with other visual techniques such as CGR and FCGR. The phylogenetic tree was constructed using MEGA6. Phylogenetic tree was constructed by the neighbor-joining method using a distance matrix from the alignment. Tree files were generated by PHYLIP and viewed using TREEVIEW program. Bootstrap analysis was also carried out to know the evolutionary history of bacteria. PCA were performed for comparative analysis [4], [5], [6], [7], [8].

### 2.1 Portal for QR codes

The QR codes of *Bacillus pumilus* were available to any user on a portal https://sites.google.com/site/bhagwanrekadwad/.

## Figures and Tables

**Fig. S1:**
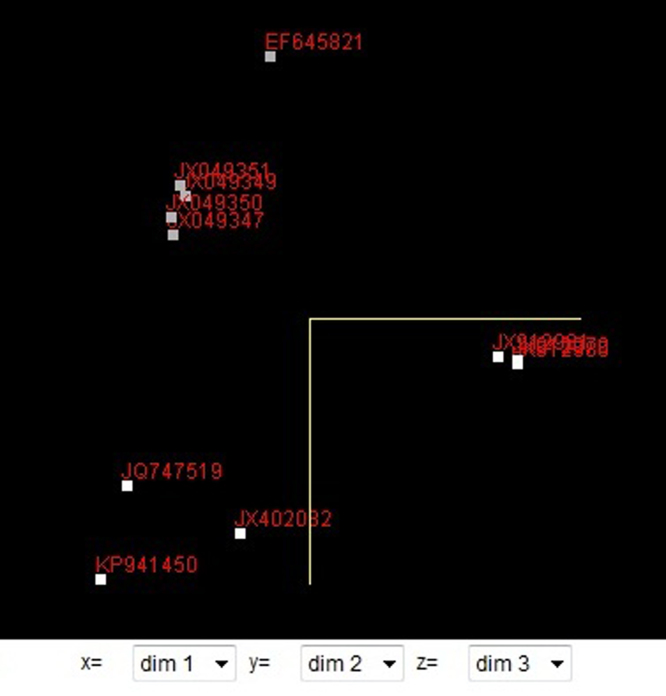
The core spectra of the chips’ surfaces after APTES and GO-COOH coating.

**Fig. S2:**
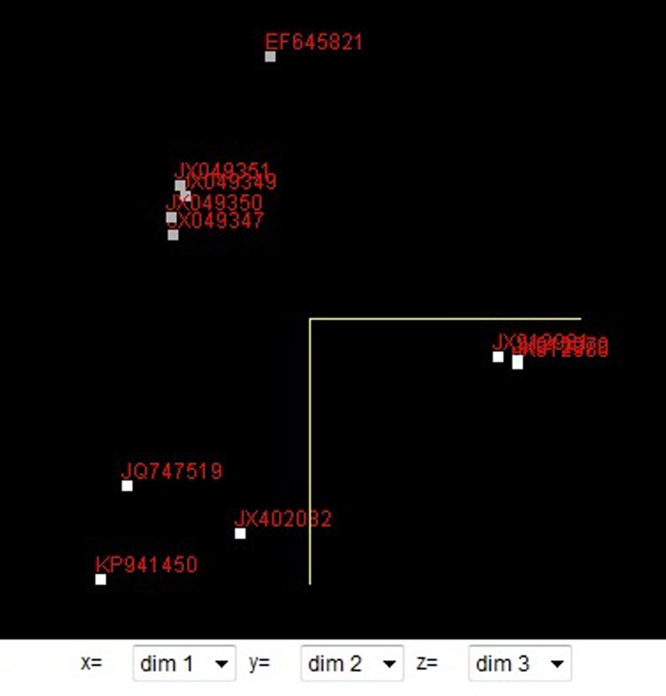
The core spectra of the chips’ surfaces after APTES and GO-COOH coating.

**Fig. S3:**
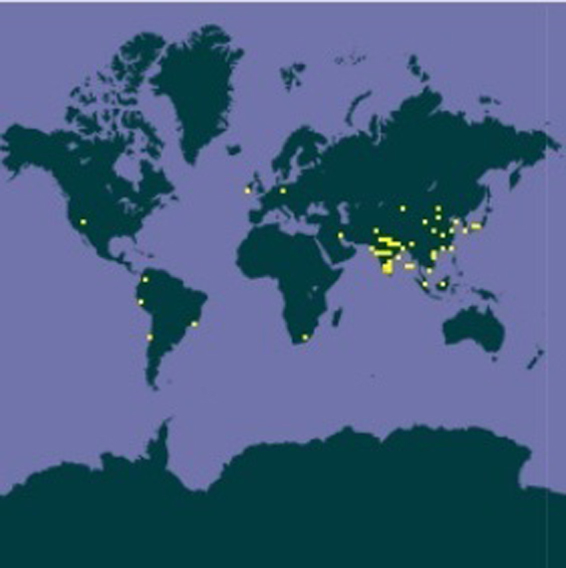
The core spectra of the chips’ surfaces after APTES and GO-COOH coating.

**Table 1:** QR codes of *Bacillus pumilus* isolated from Lonar Crator Lake, India
