# Supplementary figures and images for: Digital data for quick response (QR) codes of alkalophilic Bacillus pumilus to identify and to compare bacilli isolated from Lonar Crator Lake, India
Source: Data Brief. 2016 Apr 9;7:1306–13. doi: 10.1016/j.dib.2016.03.103 (PMC4838933; doi:10.1016/j.dib.2016.03.103)

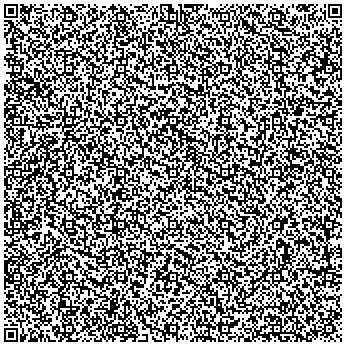

Supplement: Supplementary material [file mmc1.zip › DNABarID Full genebank QR codes/EF645821.jpg]

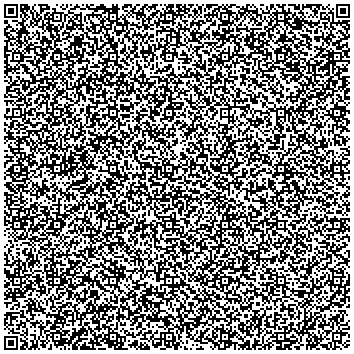

Supplement: Supplementary material [file mmc1.zip › DNABarID Full genebank QR codes/JQ747519.jpg]

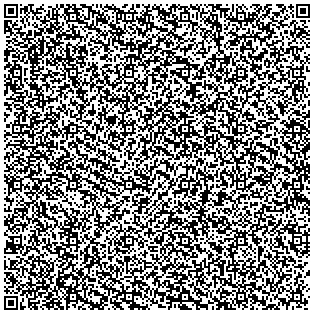

Supplement: Supplementary material [file mmc1.zip › DNABarID Full genebank QR codes/JX049347.jpg]

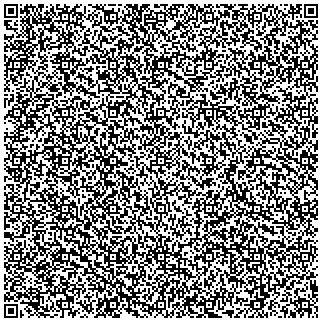

Supplement: Supplementary material [file mmc1.zip › DNABarID Full genebank QR codes/JX049349.jpg]

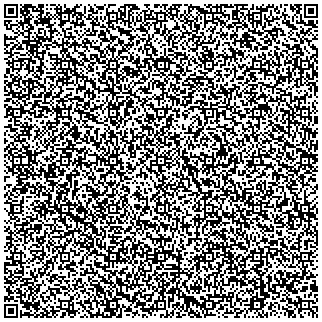

Supplement: Supplementary material [file mmc1.zip › DNABarID Full genebank QR codes/JX049350.jpg]

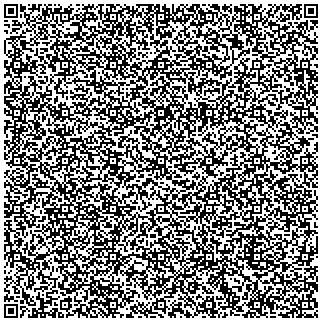

Supplement: Supplementary material [file mmc1.zip › DNABarID Full genebank QR codes/JX049351.jpg]

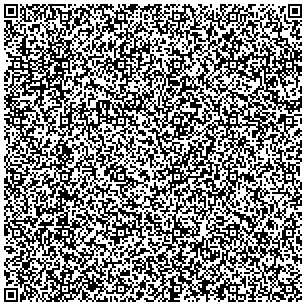

Supplement: Supplementary material [file mmc1.zip › DNABarID Full genebank QR codes/JX402082.jpg]

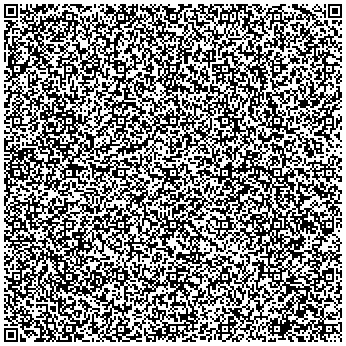

Supplement: Supplementary material [file mmc1.zip › DNABarID Full genebank QR codes/JX912979.jpg]

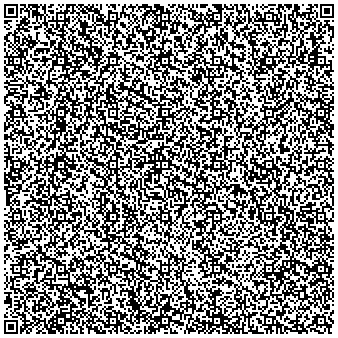

Supplement: Supplementary material [file mmc1.zip › DNABarID Full genebank QR codes/JX912980.jpg]

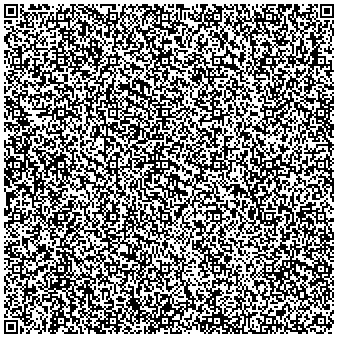

Supplement: Supplementary material [file mmc1.zip › DNABarID Full genebank QR codes/JX912981.jpg]

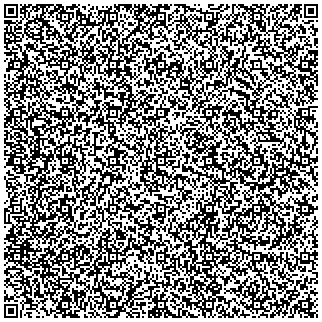

Supplement: Supplementary material [file mmc1.zip › DNABarID Full genebank QR codes/KP941450.jpg]

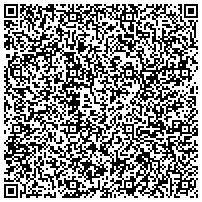

Supplement: Supplementary material [file mmc2.zip › Bacillus DNABarID/EF645821.jpg]

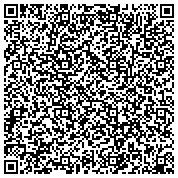

Supplement: Supplementary material [file mmc2.zip › Bacillus DNABarID/JQ747519.jpg]

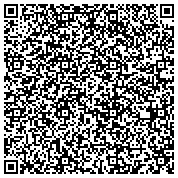

Supplement: Supplementary material [file mmc2.zip › Bacillus DNABarID/JX049347.jpg]

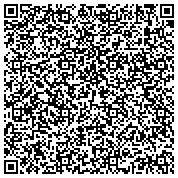

Supplement: Supplementary material [file mmc2.zip › Bacillus DNABarID/JX049349.jpg]

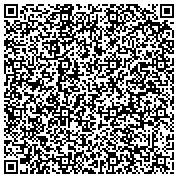

Supplement: Supplementary material [file mmc2.zip › Bacillus DNABarID/JX049350.jpg]

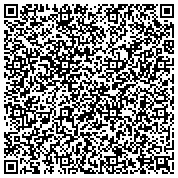

Supplement: Supplementary material [file mmc2.zip › Bacillus DNABarID/JX049351.jpg]

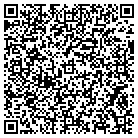

Supplement: Supplementary material [file mmc2.zip › Bacillus DNABarID/JX402082.jpg]

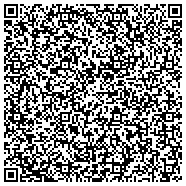

Supplement: Supplementary material [file mmc2.zip › Bacillus DNABarID/JX912979.jpg]

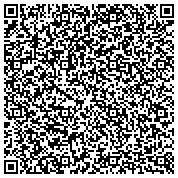

Supplement: Supplementary material [file mmc2.zip › Bacillus DNABarID/JX912980.jpg]

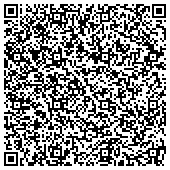

Supplement: Supplementary material [file mmc2.zip › Bacillus DNABarID/JX912981.jpg]

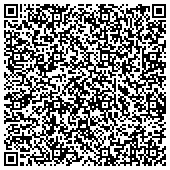

Supplement: Supplementary material [file mmc2.zip › Bacillus DNABarID/KP941450.jpg]

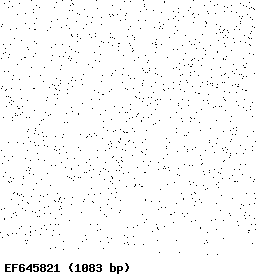

Supplement: Supplementary material [file mmc3.zip › CGR/EF645821.png]

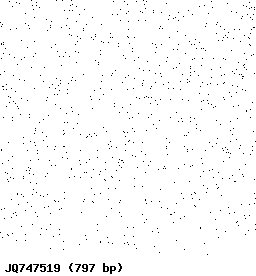

Supplement: Supplementary material [file mmc3.zip › CGR/JQ747519.png]

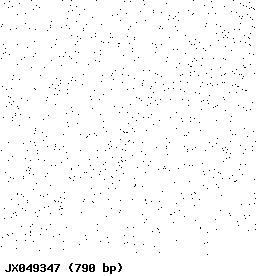

Supplement: Supplementary material [file mmc3.zip › CGR/JX049347.png]

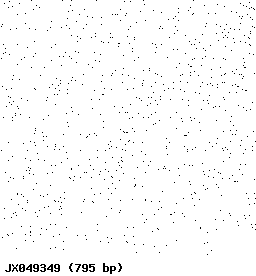

Supplement: Supplementary material [file mmc3.zip › CGR/JX049349.png]

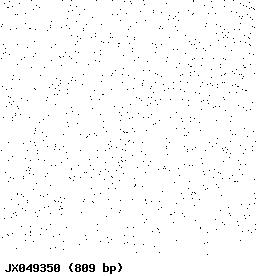

Supplement: Supplementary material [file mmc3.zip › CGR/JX049350.png]

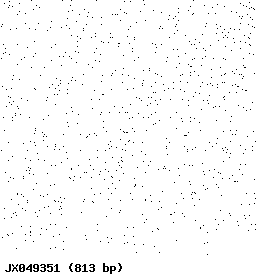

Supplement: Supplementary material [file mmc3.zip › CGR/JX049351.png]

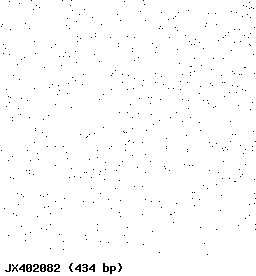

Supplement: Supplementary material [file mmc3.zip › CGR/JX402082.png]

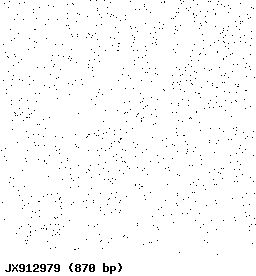

Supplement: Supplementary material [file mmc3.zip › CGR/JX912979.png]

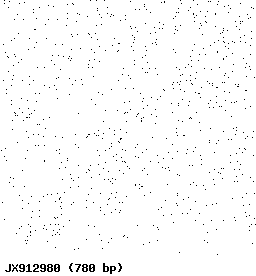

Supplement: Supplementary material [file mmc3.zip › CGR/JX912980.png]

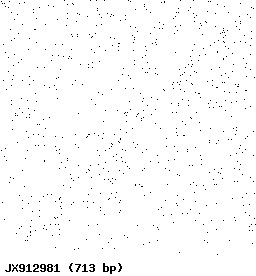

Supplement: Supplementary material [file mmc3.zip › CGR/JX912981.png]

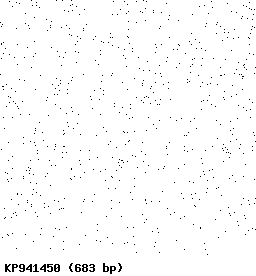

Supplement: Supplementary material [file mmc3.zip › CGR/KP941450.png]

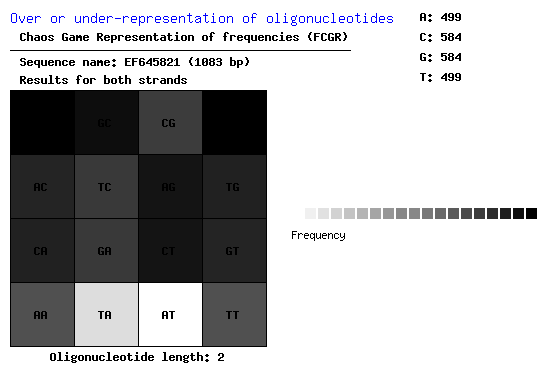

Supplement: Supplementary material [file mmc4.zip › FCGR/EF645821.png]

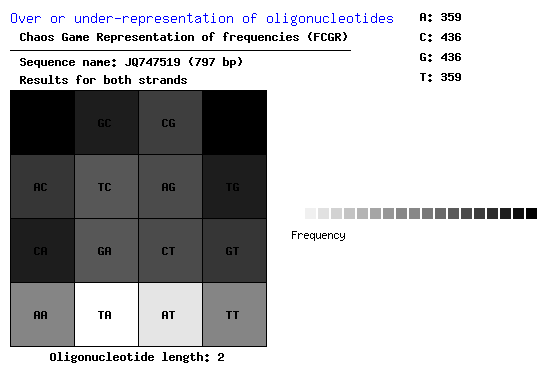

Supplement: Supplementary material [file mmc4.zip › FCGR/JQ747519.png]

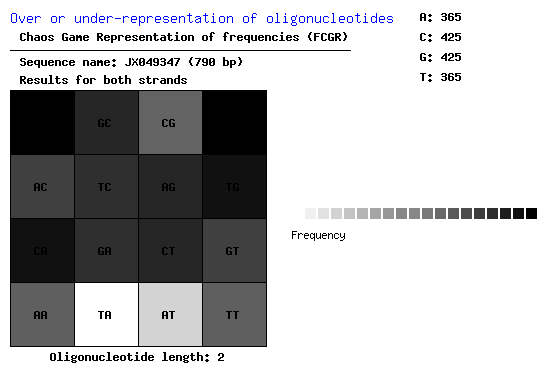

Supplement: Supplementary material [file mmc4.zip › FCGR/JX049347.png]

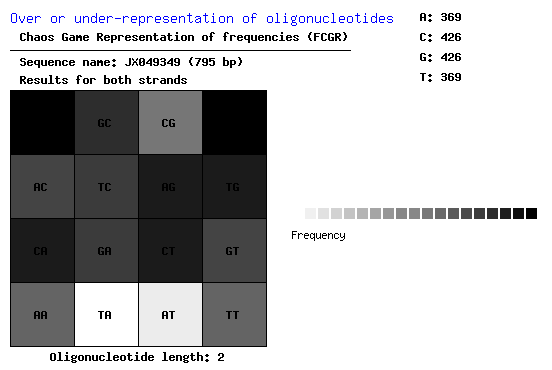

Supplement: Supplementary material [file mmc4.zip › FCGR/JX049349.png]

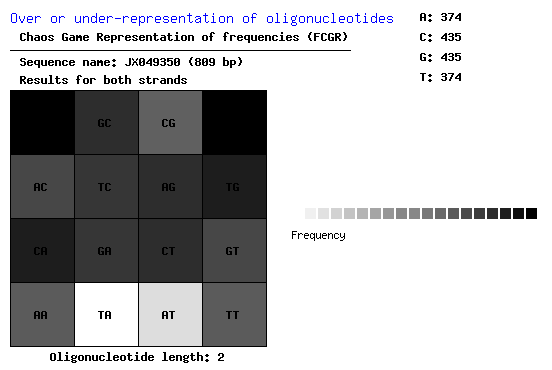

Supplement: Supplementary material [file mmc4.zip › FCGR/JX049350.png]

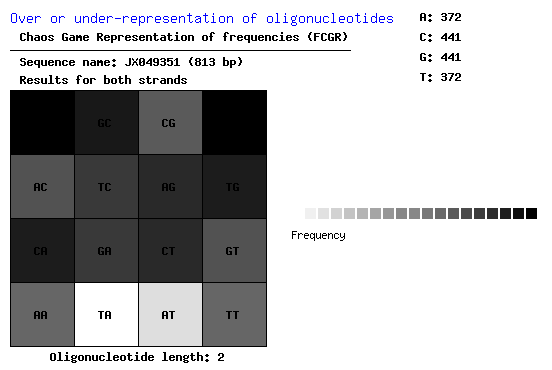

Supplement: Supplementary material [file mmc4.zip › FCGR/JX049351.png]

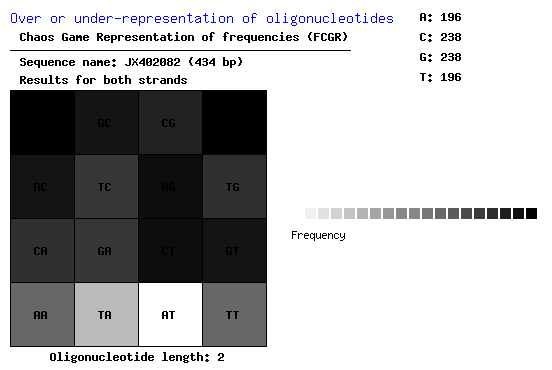

Supplement: Supplementary material [file mmc4.zip › FCGR/JX402082.png]

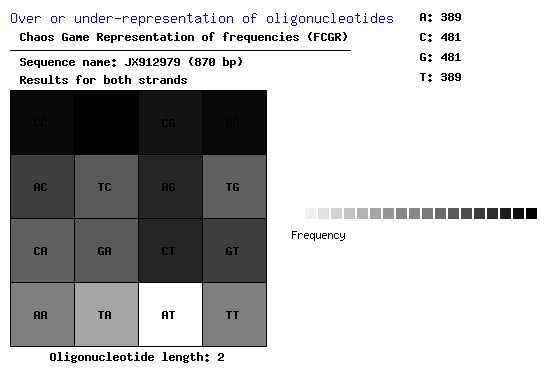

Supplement: Supplementary material [file mmc4.zip › FCGR/JX912979.png]

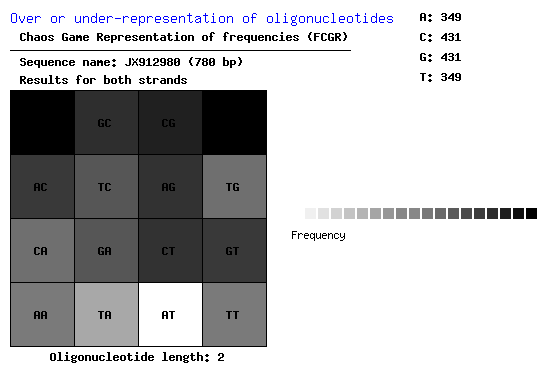

Supplement: Supplementary material [file mmc4.zip › FCGR/JX912980.png]

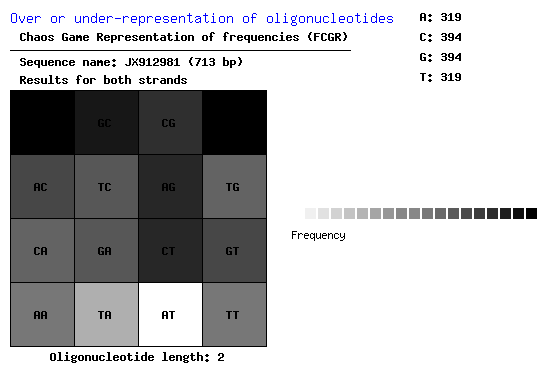

Supplement: Supplementary material [file mmc4.zip › FCGR/JX912981.png]

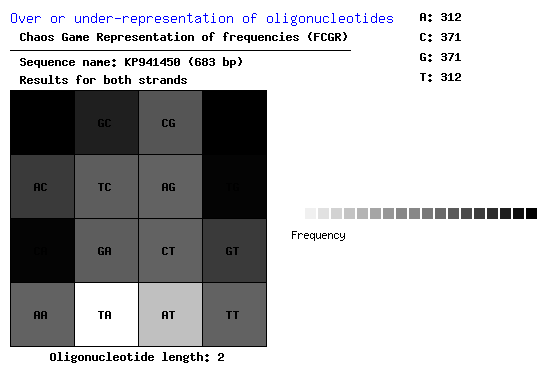

Supplement: Supplementary material [file mmc4.zip › FCGR/KP941450.png]

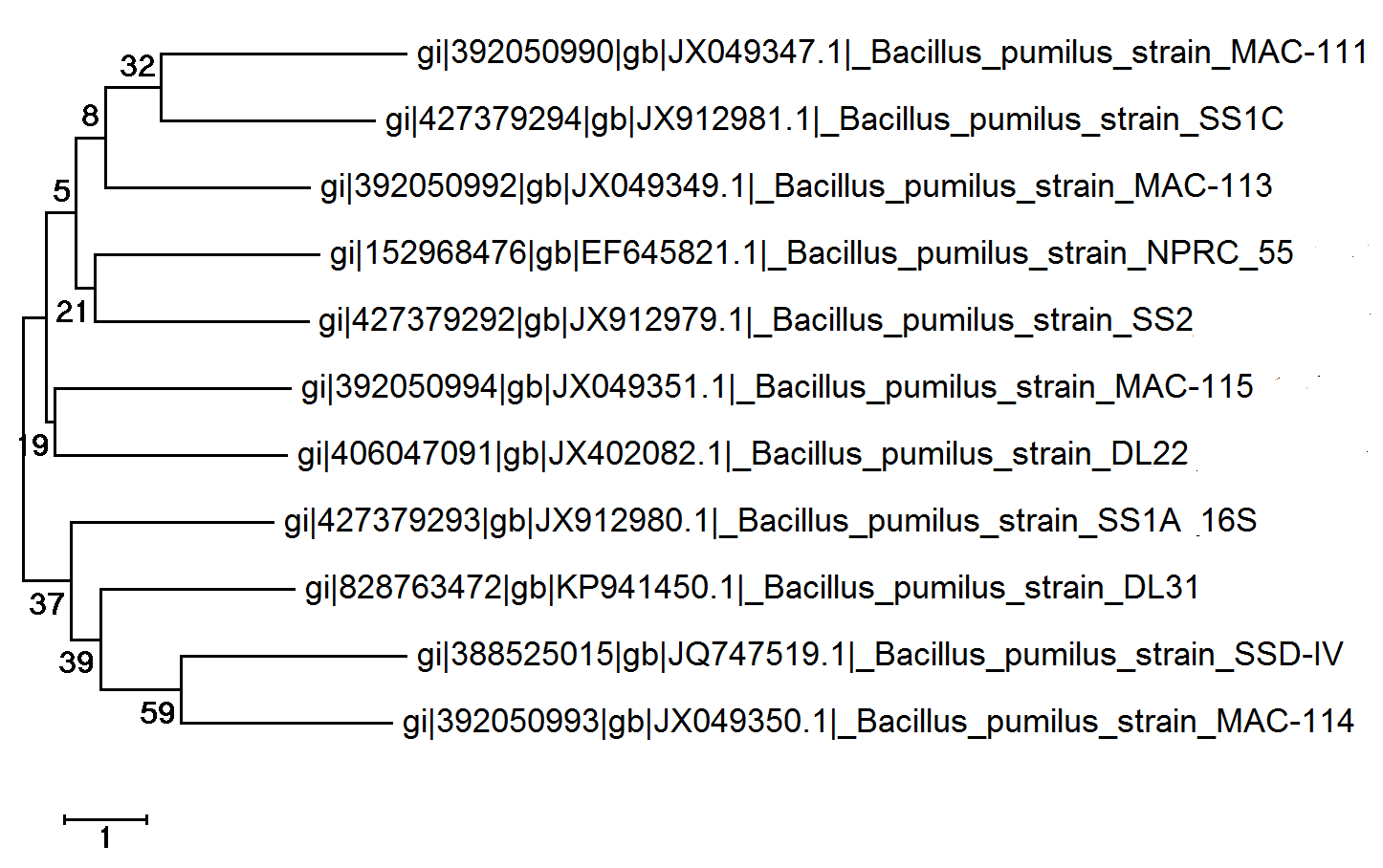

Supplement: Supplementary material [file mmc5.zip › Supplimenatary file 5_DIB_BACILLUS PUMILUS phylo tree bp.png]
